# PERK-Mediated Unfolded Protein Response Activation and Oxidative Stress in PARK20 Fibroblasts

**DOI:** 10.3389/fnins.2019.00673

**Published:** 2019-06-27

**Authors:** Giuseppina Amodio, Ornella Moltedo, Dominga Fasano, Lucrezia Zerillo, Marco Oliveti, Paola Di Pietro, Raffaella Faraonio, Paolo Barone, Maria Teresa Pellecchia, Anna De Rosa, Giuseppe De Michele, Elena Polishchuk, Roman Polishchuk, Vincenzo Bonifati, Lucio Nitsch, Giovanna Maria Pierantoni, Maurizio Renna, Chiara Criscuolo, Simona Paladino, Paolo Remondelli

**Affiliations:** ^1^Department of Medicine, Surgery and Dentistry “Scuola Medica Salernitana”, University of Salerno, Salerno, Italy; ^2^Department of Pharmacy, University of Salerno, Salerno, Italy; ^3^Department of Molecular Medicine and Medical Biotechnology, University of Naples Federico II, Naples, Italy; ^4^Section of Neuroscience, Department of Medicine, Surgery and Dentistry, University of Salerno, Salerno, Italy; ^5^Department of Neuroscience, Reproductive, and Odontostomatological Sciences, University of Naples Federico II, Naples, Italy; ^6^Telethon Institute of Genetics and Medicine, Pozzuoli, Italy; ^7^Department of Clinical Genetics, Erasmus MC, Rotterdam, Netherlands

**Keywords:** PARK20, PERK (PKR-like endoplasmic reticulum kinase), oxydative stress, ER stress, Synaptojanin 1, membrane trafficking, mitochondrial dysfunction, Parkinson’s disease

## Abstract

PARK20, an early onset autosomal recessive parkinsonism is due to mutations in the phosphatidylinositol-phosphatase Synaptojanin 1 (Synj1). We have recently shown that the early endosomal compartments are profoundly altered in PARK20 fibroblasts as well as the endosomal trafficking. Here, we report that PARK20 fibroblasts also display a drastic alteration of the architecture and function of the early secretory compartments. Our results show that the exit machinery from the Endoplasmic Reticulum (ER) and the ER-to-Golgi trafficking are markedly compromised in patient cells. As a consequence, PARK20 fibroblasts accumulate large amounts of cargo proteins within the ER, leading to the induction of ER stress. Interestingly, this stressful state is coupled to the activation of the PERK/eIF2α/ATF4/CHOP pathway of the Unfolded Protein Response (UPR). In addition, PARK20 fibroblasts reveal upregulation of oxidative stress markers and total ROS production with concomitant alteration of the morphology of the mitochondrial network. Interestingly, treatment of PARK20 cells with GSK2606414 (GSK), a specific inhibitor of PERK activity, restores the level of ROS, signaling a direct correlation between ER stress and the induction of oxidative stress in the PARK20 cells. All together, these findings suggest that dysfunction of early secretory pathway might contribute to the pathogenesis of the disease.

## Introduction

Parkinson’s disease (PD) is the second most common neurodegenerative disorder, characterized by the progressive loss of dopaminergic neurons in the substantia nigra pars compacta (Gao and Hong, 2011; Cannon and Greenamyre, 2013; Beitz, 2014; Feng et al., 2015). A combination of environmental and genetic factors has been considered to concur to the neuronal death. However, the exact molecular mechanisms are still unknown. Notwithstanding, the alteration of mitochondrial function (Winklhofer and Haass, 2010; Pilsl and Winklhofer, 2012), of reactive oxygen species (ROS) homeostasis (Gaki and Papavassiliou, 2014; Al Shahrani et al., 2017; Guo et al., 2018; Paladino et al., 2018) as well as the dysregulation of protein folding control and/or autophagic flux (McNaught and Olanow, 2006; Malkus et al., 2009; Karabiyik et al., 2017; Remondelli and Renna, 2017) have been implicated in PD pathogenesis.

Among genetic PD, PARK20 is a rare autosomal recessive juvenile Parkinson’s form due to mutations in Synaptojanin1 (Synj1), a phosphatidylinositol phosphatase (PtdInsPP) (Krebs et al., 2013; Quadri et al., 2013; Olgiati et al., 2014). The homozygous R258Q mutation was almost simultaneously reported in three unrelated families from Iran and Italy (Krebs et al., 2013; Quadri et al., 2013; Olgiati et al., 2014). Subsequently, the p.R459P mutation was found in an Indian family (Kirola et al., 2016); and, more recently, another Iranian kindred has been described with the p.R839C mutation (Taghavi et al., 2018). Finally, a frameshift mutation (p.S552Ffs^∗^5) in heterozygous state with the benign p.T1236M missense variant has been identified in one late onset PD patient from Moroccan consanguineous parents (Bouhouche et al., 2017), correlating Synj1 lesions to the risk of PD development.

Synj1 is a highly conserved PtdInsPP existing in two isoforms: the 145-kDa neuronal isoform and the ubiquitous 170-kDa isoform (Ramjaun and McPherson, 1996). Synj1 function relies on two sequential PtdInsPP domains: the N-terminal Sac1 and the central 5-phosphatase domains (5′-PP) (Di Paolo and De Camilli, 2006). The Sac1 domain of Synj1 acts on PtdIns 3- and 4-monophosphate, which are enriched in the endosomal and Golgi membranes respectively (Guo et al., 1999). Instead, the 5′-PP domain of Synj1 dephosphorylates phosphatidylinositol 4,5-bisphosphate [PtdIns(4,5)P2] located in the plasma membranes (McPherson et al., 1996; Cremona et al., 1999). Additionally, the Synj1 protein also contains a COOH-terminal proline rich region that retains the ability to interact with SH3 domains of a variety of proteins that regulate its subcellular localization and function (McPherson et al., 1996; Dittman and Ryan, 2009).

Thanks to its double enzymatic activity, Synj1 exerts multiple roles in dependence on the cell context. In nerve terminals, Synj1 participates to the control of synaptic vesicles retrieval (McPherson et al., 1996; Song and Zinsmaier, 2003; Mani et al., 2007) and cooperates with DNAJC6, another PD-causative gene (PARK19), in the process of clathrin disassembly from synaptic vesicles during endocytosis (Chang-Ileto et al., 2011; Edvardson et al., 2012). Proper Synj1 activity is essential to control homeostasis and function of early endocytic pathways in different cell types, including neuronal cells (Fasano et al., 2018). Consistently, early endosomes of PARK20 fibroblasts resulted enlarged and the recycling trafficking impaired (Fasano et al., 2018). On the other hand, unbalanced Synj1 expression is significantly involved in a number of neurological and psychiatric disorders, such as: Bipolar Disorder (Saito et al., 2001; Stopkova et al., 2004), Down’s Syndrome and Alzheimer’s Disease (Berman et al., 2008; Voronov et al., 2008; Chang and Min, 2009; Cossec et al., 2012; Martin et al., 2014), unraveling a critical role in neurons.

The p.R258Q mutation into the Synj1 Sac1 domain was shown to abolish either the 3- or 4- phosphatase activity, while it does not affect the 5-phosphatase activity (Krebs et al., 2013). Therefore, the loss of Sac1 function could alter the rate of PtdIns3P and PtdIns4P, two crucial PtdInsPs for the control of structure and function of endosomal (Efe et al., 2005; Di Paolo and De Camilli, 2006) and ER or Golgi complex membranes (De Matteis and D’Angelo, 2007; D’Angelo et al., 2008), respectively. Moreover, as we have recently shown, Sac1 domain is necessary for proper endosomal trafficking and at least 50% of its activity is required to ensure correct functionality (Fasano et al., 2018).

Here, we investigated whether p.R258Q mutation in the Sac1 domain of Synj1 could also influence vesicular trafficking at the early stages of the secretory pathway. Our experiments show that the ER exit machinery and the ER-to-Golgi trafficking are markedly compromised in p.R258Q mutated cells. As a consequence, PARK20 fibroblasts accumulate larger amounts of cargo proteins within the ER. This condition, referred to as ER stress, activates the PERK/eIF2α/ATF4/CHOP pathway of the Unfolded Protein Response (UPR) and induces oxidative stress and mitochondrial damage.

## Materials and Methods

### Cell Cultures

Fibroblasts were derived directly from the skin punch biopsies of the two Italian patients carrying the p.R258Q mutation at homozygous state (Quadri et al., 2013; Olgiati et al., 2014). A written informed consent was obtained from each patient. As control cells, primary adult Human Dermal Fibroblasts (HDF) were purchased from Sigma-Aldrich. PARK20 fibroblasts and HDF were grown in one ready-to-use Fibroblast Growth Medium (FGM from Sigma-Aldrich) at 37°C and 5% CO_2_ in humidified atmosphere. Experiments were performed on both cell lines at similar culture passages (P5-P6). When indicated, cells were starved in Fibroblasts Basal Medium (FBM from Sigma-Aldrich), which does not contain FBS and growth factors supplement. Drug treatments were performed with 1 μM GSK2606414 (Calbiochem) or 500 nM Thapsigargin (Sigma-Aldrich) for the indicated time.

### Immunofluorescence

Cells seeded on glass cover slips were washed in phosphate-buffered saline (PBS), fixed in PBS-4 % paraformaldehyde and permeabilized 30 min in PBS containing 0.5% BSA, 0.005% saponin and 50 mM NH_4_Cl. Cells were immunostained with the following primary antibodies: rabbit polyclonal anti-ERGIC-53 (α-CT) (Spatuzza et al., 2004), mouse monoclonal anti-GM130 (BD Biosciences), rabbit polyclonal anti-Giantin (Abcam), rabbit plyclonal anti-KIAA0310 (Bethyl Laboratories), rabbit polyclonal anti-Sec31a, rabbit plyclonal anti-Sar1 (Millipore), rabbit polyclonal anti-collagen IV (Rockland immunochemicals), mouse monoclonal anti-KDEL (StressGen). Primary antibodies were detected with Alexa 488- and Cy3-conjugated antibodies (Jackson Immuno Research Laboratories).

For mitochondria staining, cells were incubated for 30 min at 37°C with 200 nM Mitotracker Red CMXRos (Invitrogen-Molecular Probes) in FBM before fixing in cold acetone for 5 min on ice. Images were acquired on a laser scanning confocal microscope (TCS SP5; Leica MicroSystems or LSM 510 Meta; Zeiss MicroSystems) equipped with a plan Apo 63X, NA 1.4 oil immersion objective lens. Quantitative analysis was performed on a minimum of 30 cells by setting the same threshold of fluorescence intensity in all the samples analyzed.

Co-localization analyses and the mean intensity fluorescence quantification were carried out by using either the Leica SP5 or Zeiss software or the ImageJ program as previously described (Paladino et al., 2008; Gorrasi et al., 2014; Iorio et al., 2018; Ranieri et al., 2018). Briefly, the number of co-localized pixels was normalized for the total fluorescent pixels in the image. The degree of colocalization was assessed by calculating the Pearson’s correlation coefficient. Mean fluorescence intensity was measured in Region of Interest (ROI) of equal area in control and PARK20 samples. The number and size of SEC31a and SEC16a fluorescent spot was measured by using the ImageJ program. The distance from the nucleus of ERGIC-53 fluorescent spots was measured by using the scale bar drawing tool of Leica SP5 software.

### Electron Microscopy

Cells were fixed in 1% glutaraldehyde dissolved in 0.2 M HEPES buffer (pH 7.4) for 30 min at room temperature and then post-fixed with a mixture of 2% OsO4 and 100 mM phosphate buffer (pH 6.8) (1 part 2% OsO4 plus 1 part 100 mM phosphate buffer) for 25–30 min on ice. Then, the cells were washed three times with water and incubated with 1% thiocarbohydrizide diluted in H2O for 5 min, incubated in a mixture of 2% OsO 4 and 3% potassium ferrocyanide (1 part 2% OsO4 plus 1 part 3% potassium ferrocyanide) for 25 min on ice and overnight at 4°C in 0.5% uranyl acetate diluted in H2O. After dehydration in graded series of ethanol, the cells were embedded in epoxy resin and polymerized at 60°C for 72 hr. Thin 60 nm sections were cut at the Leica EM UC7 microtome. EM images were acquired from thin sections using a FEI Tecnai-12 electron microscope equipped with a VELETTA CCD digital camera (FEI, Eindhoven, Netherlands).

### Western Blotting

Actively growing cells seeded on 60 mm dishes were starved in FBM for 18 h prior to be subjected to the indicated treatments. Cells were then harvested in lysis buffer (10 mM Tris-HCl pH7.4, 150 mM NaCl, 1 mM EDTA pH 8.0, 1% Triton X-100) supplemented with protease and phosphatase inhibitor cocktail (Roche). Equal amounts of protein extracts were analyzed by 8 or 10% SDS-PAGE and transferred on Protran nitro-cellulose membranes (Schleicher and Schuell). Membranes were blocked either in PBS containing 10% non-fat dry milk and 0.1% Tween-20, or in TBS containing 5% BSA and 0.1% Tween-20, depending on the antibody used.

Membranes were cut in stripes according to the molecular weight expected for the single proteins analyzed, incubated with the primary followed by secondary antibodies and then visualized by ECL reaction (Amersham International) (see Supplementary Figures S3, S4). The following primary antibodies were used: rabbit monoclonal anti-PERK (Cell Signalling Technology), rabbit polyclonal anti-eIF2α and anti-phospho-eIF2α (Cell Signalling Technology), rabbit monoclonal anti-ATF4 (Abcam), mouse monoclonal anti-GADD153 (Santa Cruz Biotechnology), mouse monoclonal anti-HO1 (Santa Cruz Biotechnology) and mouse monoclonal anti-α Tubulin (Santa Cruz Biotechnology). HRP-conjugated IgG (Jackson Immuno Research Laboratories) were used as secondary antibodies. Filters were exposed to ChemiDoc MP System (Bio-Rad Laboratories Inc.) and the densitometry analysis of autoradiographs was performed by the ImageJ program on three independent experiments.

### Oxidative Stress Assays

10^6^ cells for each treatment were disposed in a well of BD Falcon 96-well black plates and starved in FBM for 18 h prior to be subjected to the indicated treatments. Cytosolic ROS were quantified by a fluorescence microplate reader [Tecan Infinite 200 Pro] using dihydrorhodamine 123 (DHR 123) probe (Santa Cruz Biotechnology), a cell-permeable non-fluorescent substance that undergoes intracellular oxidation in the presence of ROS. In detail, cells were incubated for 1 h with 50 μM of DHR123/HBSS and then washed two times with freshly prepared Hank’s balanced salt solution. Subsequently, formation of DHR 123 has been monitored by fluorescence spectroscopy using excitation and emission respectively of 500 λ nm and 536 λ nm. In some experiments, cells were pre-incubated with 1 μM GSK2606414 for 2 h, before measurements. Fluorescence signals have been recorded using Tecan i-control software and expressed as arbitrary units.

In another approach, we measured the ROS on single-cells. To this purpose, cells grown on glass bottom dishes were incubated with 2′,7′-dichlorodihydrofluorescein diacetate (DCFH-DA, 10 μM) for 10 min at 37°C in culture medium without serum and, then, imaged *in vivo* in CO_2_ independent medium as previously described (Piccoli et al., 2013). Images were collected by a Zeiss confocal LSM510 using Ar–Kr laser beam (λex 488 nm); same laser power and same settings were used for control and patient fibroblasts in all experimental conditions. Data are expressed as arbitrary units of fluorescence and reported as mean ± SD from three independent experimental conditions.

For NADPH oxidase activity measurement, the lucigenin-enhanced chemioluminescence assay was used to determine NADPH oxidase-mediated superoxide radical (O_2_^-^) production as previously described (Carrizzo et al., 2017; Schiattarella et al., 2018). Cells, cultured in 100 mm dishes, were detached using 0.25% trypsin/EDTA (1 mmol/l), washed with PBS, and resuspended in modified HEPES buffer containing (mmol/l) NaCl 140, KCl 5, MgCl2 0.8, CaCl2 1.8, Na2HPO4 1, HEPES 25 and 1% glucose, pH 7. Subsequently, cells were homogenated using VWR pellet mixer [#431-0100] and 100 μg of extract were distributed on a 96-well microplate. The reaction was started by the addition of NADPH (0.1 mmol/l) to each well (250 μl final volume) and lucigenin (5 μmol/l). The luminescence was measured using Tecan Infinite M200 multimode microplate fluorometer at 37°C every 10 s for 60 min. Each experiment was performed in triplicate. In some experiments, cells were pre-incubated with 1 μM GSK2606414 for 2 h, before measurement of luminescence.

### RT-PCR and XBPI Splicing Assay

One microgram of DNAse-treated total RNA was retro-transcribed with the Easy-script plus cDNA synthesis Kit (abm) according to manufacturer instructions. Semi-quantitative PCR was performed on 3 μl of cDNA with the following primers Bip/Grp78-forward: 5′-CTG GGT ACA TTT GAT CTG ACT GG-3′; Bip/Grp78-reverse: 5′-GCA TCC TGG TGG CTT TCC AGCCAT TC-3′; GAPDH-forward: 5′-GAA GGT GAA GGT CGGAGT C-3′; GAPDH-reverse: 5′-GAA GATGGT GAT GGG ATTTC-3′ (Amodio et al., 2011). XBPI splicing assay was performed as previously described (Eletto et al., 2016) by using the following primers: 5′-A AAC AGA GTA GCA GCT CAG ACT GC-3′ and 5′-C CTT CTG GGT AGA CCT CTG GGA G-3′. The resulted un-spliced and spliced XBP1 mRNA were separated by gel electrophoresis on 3% agarose gel. Ethidium bromide-stained amplicons were quantified by densitometry with ImageJ software.

### Statistical Analysis

Data are expresses as mean ± SD. All statistical analyses using Student’s *t*-test and histograms were completed with Prism statistical software (Graphpad, La Jolla, CA, United States) and differences were considered statistically significant when *P* < 0.05.

## Results

### PARK20 Fibroblasts Show Unbalanced ER-to-Golgi Trafficking and Abnormal Structure of Golgi Membranes

To test whether membrane trafficking from the ER to the Golgi complex was affected by the p.R258Q mutation, we analyzed fibroblasts derived from homozygous R258Q/R258Q PARK20 patients and from healthy individuals.

**FIGURE 1:**
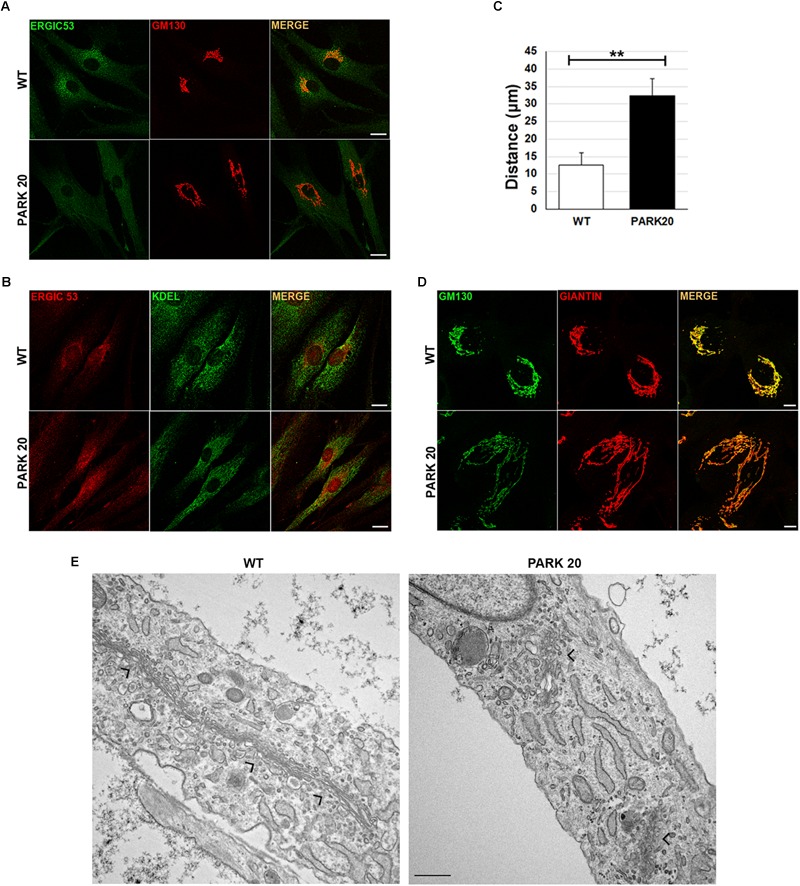
Protein markers of the early secretory pathway are differently distributed in WT and PARK20 fibroblasts. **(A,B,D)** HDF (WT) and PARK20 cells were seeded on glass coverslips, fixed and processed for immunofluorescence with the indicated antibodies. Images were collected by confocal microscope. Scale bars: 10 μm. **(C)** Histogram shows the distance (mean ± SD in μM) from the nucleus of ERGIC-53 fluorescent spots. *N* ≥ 30. ^∗∗^*p* ≤ 0.01, Student’s *t*-test. **(E)** Representative ultrastructural images of WT and PARK20 cells. Scale bar: 1 μm; arrows indicate the position of Golgi complex.

Dynamics of membrane trafficking at the early steps of the secretory pathway were analyzed by looking at the intracellular distribution of vesicles carrying the cargo receptor ERGIC-53 (Appenzeller et al., 1999). Normally, the ERGIC-53 protein cycles between the ER and the Golgi complex (Appenzeller et al., 1999) and ERGIC-53 containing vesicles show their typical punctuate distribution depicted by higher concentration in the region closed to the *cis*-Golgi membranes, which in turn are labeled with the resident protein GM130 in wild-type cells (Figure 1A, wt). Instead, in patient fibroblasts ERGIC-53 vesicles were reduced both in size and fluorescence intensity (Figure 1A, PARK20). In addition, they are delocalized throughout the cytoplasm at higher distance from the perinuclear region with a mean value of 12.3 ± 3.4 μm in the PARK20 cells vs. 31.8 ± 4.3 μm in the control cells (Figure 1C). Interestingly, ERGIC53 vesicles redistribution pattern in the PARK20 cells overlapped with the membrane network of the ER, as shown by the fluorescence detected by the anti-KDEL antibody, which label ER resident proteins bearing the KDEL retrieval sequence (Figure 1B). In addition, we also detected dramatic changes in the organization of Golgi membranes of PARK20 fibroblasts (Figure 1A,D). Both the *cis*-Golgi membranes labeled by the resident protein GM130 (Figure 1A) and the overall Golgi architecture revealed by the structural Golgi protein giantin (Figure 1D) were more dispersed and relocated in tubular structures extending from the nucleus to the cell edge in the PARK20 cells with respect to control (Figure 1A,D). The ultrastructural analysis further showed that the Golgi complex is scattered throughout the cell in PARK20 fibroblasts (Figure 1E, arrows). Moreover, while GM130 co-localized almost completely with giantin in control cells, they resulted partially co-distributed in patient cells (Figure 1D).

Since both GM130 and giantin are involved in the ER-to-Golgi trafficking (Alvarez et al., 2001), these results further suggest that PARK20 cells undergo unbalanced trafficking at the early steps of the secretory pathway.

### PARK20 Fibroblasts Show Reduced Formation of COPII Carrier Vesicles

**FIGURE 2:**
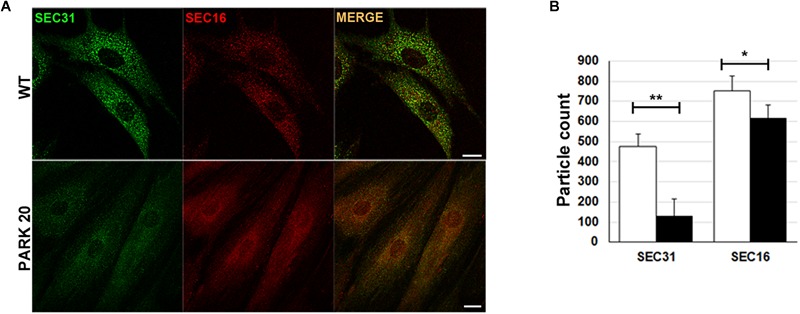
The formation of COPII-coated vesicles are reduced in PARK20 fibroblasts. **(A)** HDF (WT) and PARK20 cells seeded on glass coverslips were fixed, stained with the indicated antibodies and analyzed by confocal immunofluorescence. Scale bars: 10 μm. **(B)** Histogram shows particle count (mean ± SD) of Sec31 (green) and Sec16 (red) fluorescent spots analyzed by Image J. *N* ≥ 30. ^∗^*p* ≤ 0.05, ^∗∗^*p* ≤ 0.01, Student’s *t*-test.

The results described above prompted us to test whether the abnormal organization of post-ER compartments observed in the PARK20 cells was the result of reduced flow of carrier vesicles budding from the ER. Formation of transport vesicles from the ER membranes requires the assembly of the vesicular coat (COPII) at specific ER membrane domains defined ER exit Sites (ERESs), recognized by the presence of the endogenous Sec16 protein (isoform A). As a rule, Sec16 recruits COPII components for their assembly at the ERESs (Watson et al., 2006). As expected, these latter are visible as punctuate structures dispersed throughout the cytoplasm (Figure 2A, Sec16/wt). Instead, in PARK20 cells the number of puncta of Sec16 fluorescence are reduced (Figure 2A,B; Sec16/PARK20), indicating that the number of ERESs is considerably decreased in PARK20 cells. As a consequence, the number of COPII vesicles, revealed by antibody recognizing Sec31 (Figure 2A, Sec31/wt), a component of the outer layer of the COPII vesicles, was also reduced (Figure 2A,B; Sec31/PARK20). Moreover, Sec16 co-localized with Sec31 at the same extent as in control cells (Figure 2A, merge), suggesting that Sec16 still organizes COPII assembly at ERESs, but with less efficiency.

Thus, our results strongly indicate that the Synj1 activity localized in the Sac1 domain of the protein is essential for the proper function of COPII. In particular, the reduced number of ERES found in the PARK20 cells indicates that altered phosphatase activity of the Synj1 Sac1 domain reduces the amount of ER exit sites, thus biasing the assembling and/or the stability of COPII vesicles.

### The Secretion of Collagen IV Is Impaired in PARK20 Fibroblasts

**FIGURE 3:**
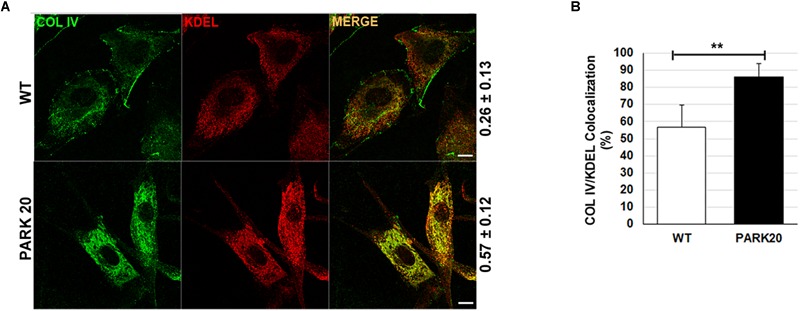
The secretion of collagen IV is impaired in PARK20 fibroblasts. **(A)** HDF (WT) and PARK20 cells were seeded on glass coverslips, processed for immunofluorescence and analyzed by confocal imaging. Numbers on the right refers to Pearson’s correlation coefficient, quantified with Leica SP5 software (mean ± SD). *p* ≤ 0.01, Student’s *t*-test; *N* ≥ 30; scale bars: 10 μm. **(B)** Histogram shows the percent of collagen IV pixels colocalizing with KDEL pixels (mean ± SD) analyzed by Leica SP5 software. ^∗∗^*p* ≤ 0.01, Student’s *t*-test.

To test whether reduced formation of ERESs could have an effect on the rate of cargo proteins transport from the ER to the Golgi complex, we examined the level of distribution of endogenous collagen IV (COLIV) along the secretory pathway in PARK20 cells vs. control cells (Figure 3A). Typically, in normal fibroblasts COLIV is secreted from the cell and accumulates in structures located in the interstitial space out of the cell (Figure 3A, COLIV/wt) (Amodio et al., 2016). In PARK20 fibroblasts, COLIV was mainly found within intracellular membranes resembling the ER network stained by the KDEL antibody (Figure 3A: compare PARK20 pro-COLIV to KDEL), indicating that COLIV secretion is reduced. In PARK20 fibroblasts, COLIV did not accumulate outside the cell (Figure 3A, PARK20), but it was mainly found within intracellular membranes, in structures that resemble the ER network stained by the KDEL antibody (Figure 3A: compare PARK20 pro-COLIV to KDEL). Consistently, by analyzing the rate of colocalization with ER resident proteins labeled by the anti-KDEL antibody, we found an increase in procollagen IV (pro-COLIV) colocalization with ER membranes: 87.1 ± 7.7% in the Synj1 mutated cells compared to 56.6 ± 13.3% in control cells (Figure 3B). In line with the immunofluorescence assays, we found that secretion of COLIV by PARK20 fibroblasts into culture medium was strongly reduced in comparison to control fibroblasts (Supplementary Figure S1A). All these data indicate that secretion of COLIV in the PARK20 cells was almost completely inhibited presumably as a consequence of the reduced function of the ER exit machinery. Moreover, a slight but appreciable reduction of total protein secretion is observed in PARK20 fibroblasts as well as a reduction of total delivery to the surface (Supplementary Figure S1), all suggesting an impairment of secretory trafficking.

### The PERK/eIF2a/ATF4 Branch of the Unfolded Protein Response Is Activated in PARK20 Fibroblasts

**FIGURE 4:**
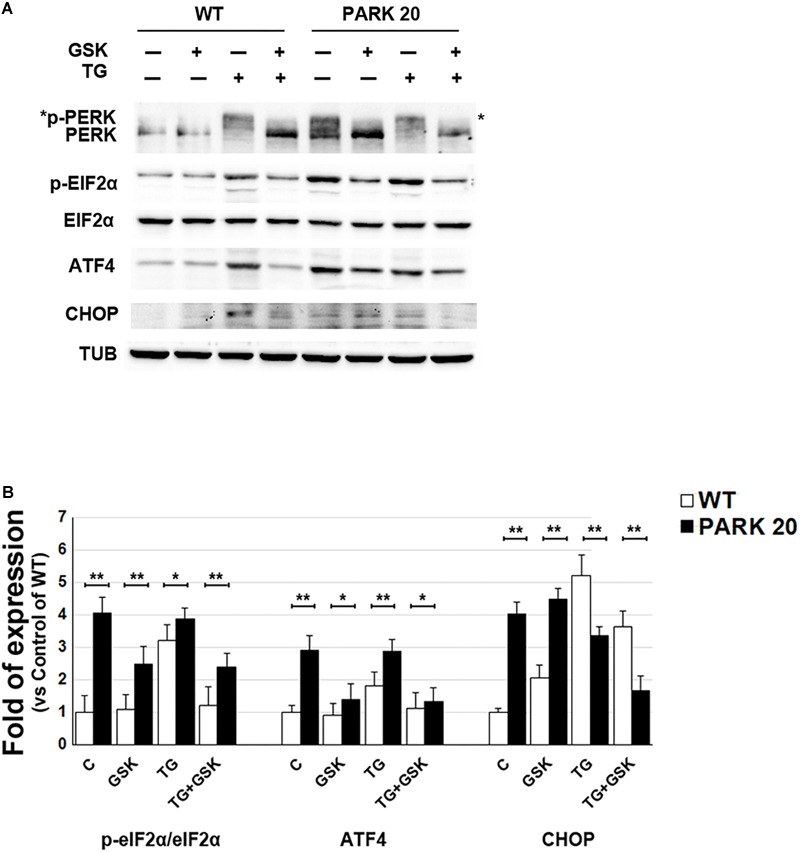
The PERK-dependent pathway of the UPR is up-regulated in PARK20 fibroblasts. **(A,B)** Western blot analysis of the relative expression of markers of the PERK-dependent pathway of UPR. Starved HDF (WT) and PARK20 cells were either untreated (C) or treated for 2 h with 1 μM GSK2606414 (GSK) or 500 nM Thapsigargin (TG) alone (GSK and TG respectively) or in combination (TG + GSK). For TG + GSK samples, a pre-treatment of 30 min with GSK2606414 was performed prior to the addition of TG. α-Tubulin (TUB) was used as loading control. **(B)** Densitometry analyses of three independent experiments are shown. Histogram shows the relative fold induction of the expression of the indicated proteins in the treated samples compared to the control samples. Results are expressed as mean values ± SD; ^∗^*p* ≤ 0.05, ^∗∗^*p* ≤ 0.01, Student’s *t*-test.

The presence of higher amounts of proteins retained into the ER, prompted our investigation into whether such accumulation could activate the ER stress and, as a consequence, the UPR (Walter and Ron, 2011). Therefore, we analyzed the activation state of components of the UPR signaling, such as PERK and IRE1, and the expression level of typical marker of ER stress (Franceschelli et al., 2011; Hiramatsu et al., 2011). To determine PERK activation, we analyzed by western blotting the phosphorylated PERK form (p-PERK) and eIF2α form (p-eIF2a) expressed in the cell extracts obtained from control and PARK20 fibroblasts (Figure 4). In particular, PERK phosphorylation was recognized throughout immunoblots (Figure 4A) showing the band-shift of p-PERK in western blot analyses as a consequence of the higher molecular weight acquired by the auto-phosphorylation (Harding et al., 1999; Eletto et al., 2016). As expected, in control cells we did not detect p-PERK form in basal conditions, but only after treatment with the UPR inducer thapsigargin (TG) (Figure 4A). Strikingly, p-PERK was highly detectable in uninduced PARK20 cells, suggesting that the PERK branch of the UPR was constitutively turned on in PARK20 fibroblasts (Figure 4A). As in control fibroblasts, cell exposure to the PERK inhibitor GSK abolished kinase activity of the PERK protein (Figure 4A), confirming that this pathway is activated in Synj1 mutated cells.

Then, we analyzed the phosphorylation status of elF2α by using a p-eIF2α antibody, which specifically detects the phosphorylated form of the protein (Figure 4A) and we found higher levels of p-eIF2α in PARK20 fibroblasts with respect control cells (Figure 4A,B). Moreover, PERK inhibitor GSK was able to reduce p-eIF2α amount in the Synj1 mutated cells (Figure 4A,B).

Finally, since increased eIF2α phosphorylation induces the ATF4/CHOP pathway of the ER stress, we analyzed the expression of ATF4 and CHOP in the PARK20 cells and found that both proteins enhanced with respect to control cells (Figure 4A,B). Moreover, after PERK inhibitor incubation ATF4 significantly reduced. Conversely, CHOP was not influenced by GSK, suggesting the involvement of positive feedback loops activated downstream to PERK induction (Figure 4A,B).

We also tested whether in the Synj1 mutated cells, the IRE1 arm of the UPR and/or the expression of genes under the transcriptional control of the ATF6 pathway of the UPR were equally activated. We found that either the IRE1 endonuclease activity or expression levels of ATF6-controlled genes in the PARK20 cells were similar to those detected in control cells (Supplementary Figure S2).

In summary, our results reveal that in the PARK20 cells the PERK/eIF2α/ATF4 pathway of the UPR is constantly activated, presumably as a result of the persistent activation of ER stress induced by the overload of cargo protein within the ER.

### Persistent Activation of the PERK Pathway of the UPR Induces Oxidative Stress in PARK20 Cells

**FIGURE 5:**
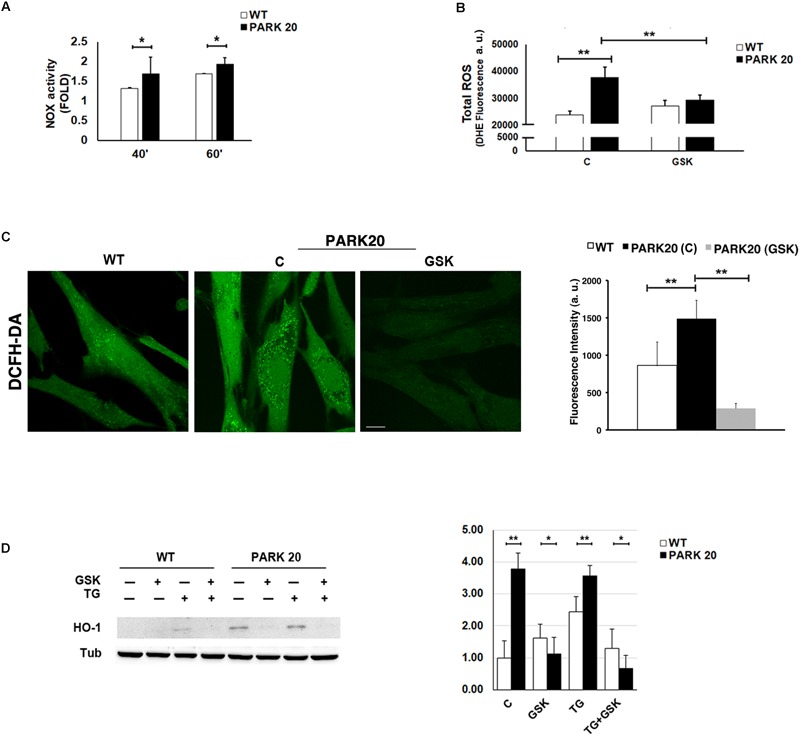
Oxidative stress is increased in PARK20 fibroblasts. **(A)** NADPH oxidase (NOX) activity assay performed in HDF cells (WT) and PARK20 fibroblasts as reported in the methods. Histogram shows the relative fold change of NOX activity expressed as mean ± SD. Results were obtained from three independent experiments. **(B,C)** ROS quantification was carried out by using DHE **(B)** or DCFH-DA **(C)** fluorescent probes. HDF cells (WT) and PARK20 fibroblasts were untreated (C) or treated for 2 h with 1μM GSK2606414 (GSK) and then processed for DHE or DCFH-DA fluorescence quantification as detailed in the methods section. Histograms show mean values ± SD of DHE or DCFH-DA fluorescence expressed as arbitrary unit (a. u.) and calculated on three independent experiments. Scale bar 10 μm. **(D)** Immunoblot detection of HO-1 and densitometric analysis of three independent experiments. Starved HDF (WT) and PARK20 cells were either untreated (C) or treated for 2 h with 1 μM GSK2606414 (GSK) or 500 nM Thapsigargin (TG) alone (GSK and TG respectively) or in combination (TG + GSK) as aforementioned. α-Tubulin (TUB) was used as loading control. Representative immunoblotting is shown. Histogram shows the relative fold induction of HO-1 expression in the treated samples compared to the control samples. Results are expressed as mean values ± SD. ^∗^*p* ≤ 0.05, ^∗∗^*p* ≤ 0.01, Student’s *t*-test.

The alteration of ER proteostasis and the consequent accumulation of misfolded proteins within the ER is associated with the increment of ER protein folding that strongly induces ROS production (Tu and Weissman, 2004; Santos et al., 2009). Since NADPH oxidase is one of the key sources of cytosolic ROS (Lambeth, 2004), we measured the activity of the NADPH oxidase (NOX) through a quantitative lucigenin-based luminescence assay. Higher NOX activity was observed in PARK20 cells compared to control cells at each time point (Figure 5A), demonstrating that PARK20 fibroblasts exhibited pronounced oxidative stress.

It is well documented that the UPR could modulate the oxidative state of the cell, in particular through the PERK/eIF2α/ATF4 pathway (Amodio et al., 2018). Therefore, given the activation of the PERK pathway found in PARK20 fibroblasts, we measured the levels of cytosolic Reactive Oxygen Species (ROS) by dihydroethidium (DHE) fluorescent probe, in absence or in presence of the PERK inhibitor GSK (Figure 5B). Upon basal conditions, a significant higher amount of ROS production was detected in PARK20 cells with respect to control cells (24000 vs. 38000 a. u.; 1.6 Fold) (Figure 5B). Interestingly, GSK treatment reduced drastically ROS-derived DHE fluorescence, retrieving it to the values found in the control cells (Figure 5B). Alternatively, ROS levels were assessed by confocal microscopy imaging cells with the redox-sensitive fluorescent probe 2′,7′ dichlorodihydrofluorescein diacetate (DCFH-DA) obtaining similar results (Figure 5C).

All these data indicate that PERK inhibition significantly reduces cytosolic ROS generation, thus providing evidence that the activation of the PERK pathway of UPR is responsible for the induction of oxidative stress in PARK20 fibroblasts.

Because the PERK pathway of the UPR can also activate antioxidant factors, such as the neuroprotective haemeoxygenase-1 (HO-1) enzyme (Alam et al., 1999; Cullinan et al., 2003; Kensler et al., 2007), we analyzed the expression level of the HO-1 protein in control and PARK20 fibroblasts (Figure 5D). Our results clearly show a consistent up-regulation and PERK-dependant expression of the HO-1 protein in PARK20 cells, suggesting that PERK activation could also induce an antioxidant response to oxidative stress in PARK20 cells (Figure 5D).

**FIGURE 6:**
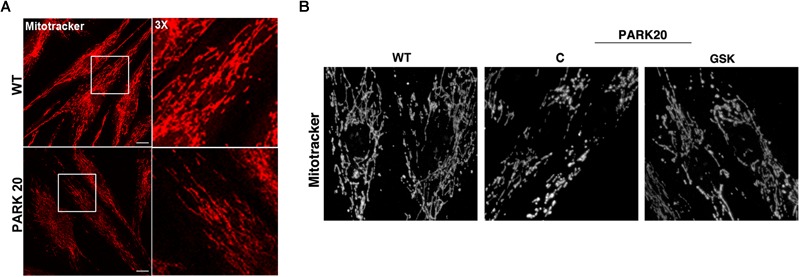
Mitochondrial network is altered in PARK20 fibroblasts. HDF (WT) and PARK20 cells were seeded on glass coverslips and stained with the mitotracker red CMXRos probe for 20 min at 37°C prior to be fixed and processed for immunofluorescence. Serial confocal sections were collected from top and bottom of the cells. Representative images of single confocal sections **(A)** and 3D reconstructions of Z-stack acquired to higher magnification **(B)** are shown. Scale bars: 20 μm.

Furthermore, because mitochondria represent the major source of intracellular ROS and their dysfunction is widely reported in PD (Balaban et al., 2005; Lin and Beal, 2006), we investigated whether the observed cytosolic ROS overproduction is associated with mitochondria impairment. Because mitochondrial function is linked to the overall organization of the mitochondrial network (Chan, 2012), we examined the mitochondrial morphology by using the Mitotracker Red CMXRos probe (Figure 6). The morphology of mitochondrial network is profoundly altered in PARK20 fibroblasts compared to control cells (Figure 6). As evident by 3D reconstructions in the patient cells, mitochondrial network lost the typical interconnected tubular structure and exhibited thinner and shorter tubules with many fewer branch points (Figure 6B). Moreover, we also observed a reduced Mitotracker staining in PARK20 compared to control cells (Figure 6A). Considering that Mitotracker probe accumulates in mitochondria depending on its membrane potential (MMP), the reduced staining suggests a loss in MPP.

All these data indicate that, in addition to the increase in cytosolic ROS, in the PARK20 cells mitochondrial alteration was dependent on PERK activation and quite reversed by the PERK inhibitor GSK2606414 (Figure 6B).

## Discussion

In the present work we investigated further into the molecular events underlying the biogenesis of the juvenile parkinsonism PARK20, highlighting a role of Synj1 in regulating the early steps of secretory pathway.

Previous studies have well established that Synj1 is a crucial player for synaptic vesicle endocytosis and renewal at the nerve terminals, thanks to its 5-phospatase activity (Cremona et al., 1999; Kim et al., 2002; Mani et al., 2007; Cao et al., 2017). Recently, it has been demonstrated that Synj1 controls endosomal trafficking in different cell types including neuronal cells, presumably by regulating the of PtdIns3P levels within the endocytic pathways (Efe et al., 2005; Di Paolo and De Camilli, 2006; Krebs et al., 2013; Fasano et al., 2018).

Our results reveal a novel role for Synj1, consisting in the regulation of membrane trafficking at the ER-to-Golgi boundaries. It is likely that this regulatory function might be associated to its PtdIns4P phosphatase activity since ER and Golgi membranes are enriched in this phosphoinositide and the PtdIns4P phosphatase activity of Synj1 is affected by R258Q mutation (Krebs et al., 2013). Consistently, similar effect can be observed when persistent decrease of PtdIns4P hydrolysis is generated by deletion of the PtdIns4P phosphatase Sac1, the major lipid 4-phosphatase in yeast and mammals (Foti et al., 2001; Tahirovic et al., 2005; Liu et al., 2008).

Interestingly, by the use of a newly developed high-resolution imaging technology Sac1, which has been shown to shuttle between ER and Golgi in response to different stimuli (Blagoveshchenskaya and Mayinger, 2009), has been recently found to reside at the ER-Trans Golgi Network (TGN) contacts sites, where its phosphatase activity controls either ER or Golgi PtdIns4P membrane content (Venditti et al., 2019a,b). ER can establish membrane contacts with TGN as well as with endosomal membranes, referred to as ER-endo-lysosomal contact sites (EELCS) (Eden, 2016; Henne, 2017).

Given the interconnection of Synj1 with the endolysosomal pathway, an interesting hypothesis, to be tested in future work, would be to establish, whether Synj1 resides at EELCS and how EELCS helps Synj1/Sac1 activity to maintain PtdIns4P homeostasis.

Another finding supporting a role of Synj1 in controlling the early steps of secretory pathway is the altered distribution of GM130 and giantin, two Golgi factors also involved in the ER-to-Golgi trafficking (Alvarez et al., 2001). Furthermore, PARK20 cells display dramatic changes in the organization of Golgi membranes. The loss of intact Golgi populations with concomitant increase in vesiculated and dispersed Golgi membranes might be due to the unbalanced trafficking or defective membrane tethering and fusion events, consequent to the loss of Synj1 PtdIns4P phosphatase activity (Foti et al., 2001; Tahirovic et al., 2005; D’Agostino et al., 2014, 2018).

Central finding of our work is the discovery that formation of carrier vesicles at the endoplasmic reticulum exit sites (ERESs) is largely inhibited. The consequent traffic jam of secretory proteins within the ER membranes generates the activation of the PERK pathway of the ER stress/UPR, which in turn induces oxidative stress and mitochondrial damage. Therefore, the primary event causing the ER stress activation might rely on the altered control of the PtdIns4P content at ER membranes with consequent impairment of carrier vesicles formation. Indeed, the dynamic control of PtdIns4P level is necessary to coordinate the progression of both ERES formation and COPII assembly (Nagaya et al., 2002; Pathre et al., 2003; Blumental-Perry et al., 2006; Farhan et al., 2008). In these events, a specific role is played by p125A that, upon PtdIns4P recognition, promotes the recruitment of Sec16 at the ERESs, which in turn favors COPII assembly and cargo export from the ER (Shimoi et al., 2005; Iinuma et al., 2007; Ong et al., 2010).

Our results indicate that loss of Sac1/Synj1 activity in the R258Q/R258Q PARK20 cells could profoundly alter these dynamic events, leading to defective export of secretory proteins from the ER.

In PARK20 cells the PERK/eIF2α/ATF4 pathway of the UPR is hyperactive in response to the persistent state of ER stress induced by the ER overload of cargo proteins. This finding opens a novel perspective in the understanding of the molecular events leading to the PARK20 phenotype. The activation of the PERK pathway of the UPR is a common hallmark of various neurodegenerative diseases. In particular, as shown from postmortem analyses in PD patients as well as in animal models of PD, the activation of the PERK pathway represents a common cause of death of dopaminergic neuron (Scheper and Hoozemans, 2015; Gully et al., 2016). In our case, we show that, in PARK20 cells, prolonged PERK activation generates a pronounced production of cytosolic ROS, whereas GSK-mediated inhibition of PERK drastically reduces ROS production.

In this regard, it is worth noting that UPR, through multiple different pathways, can give rise to either pro-oxidant or antioxidant response (Malhotra and Kaufman, 2007; Amodio et al., 2018). In particular, during ER stress the increment of ER protein folding demand strongly induces ROS production (Tu and Weissman, 2004; Santos et al., 2009). In this context, PERK can play the role of a double-edged sword. In fact, in first instance, the PERK-eIF2α-ATF4 axis operates to restore ER proteostasis by reducing ER protein load and by inducing antioxidant pathways through the activation of the transcription factor nuclear factor erythroid 2-related factor 2 (NRF2) (Alam et al., 1999; Cullinan et al., 2003; Kensler et al., 2007). On this line, we found a significant up-regulation of HO-1 expression in PARK20 cells. On the other hand, the persistent activation of PERK in condition of unsolved protein misfolding can boost up ROS production and induce factors of the pro-oxidant signaling pathway. Among these, the transcription factor CHOP, activated downstream to the PERK/eIF2α/ATF4/CHOP pathway, plays an important role. CHOP promotes the expression of both Ero1 and NOX that are responsible for ROS production during the oxidative protein folding and ER stress (Li et al., 2010; Anelli et al., 2012). Accordingly, we found a significant increased expression of CHOP in PARK20 cells besides the reported increase of cytosolic ROS. In this regard, it is worth remarking that normal CHOP expression is not recovered after GSK treatment. This finding is not surprising, since after its PERK-dependent activation, CHOP activates other downstream pathways that positively feedback on CHOP expression. Additionally, it is important to consider that CHOP triggers the ERo1-IP3R1-Ca2+/calmodulin-dependent protein kinase II (CaMKII)-NOX2 cascade, in which NOX2 finally induces CHOP expression in a manner independent by PERK (Li et al., 2010; Anelli et al., 2012). All in all, our data support co-existence of a PERK-dependent pro-oxidant and anti-oxidant response. However, we do not know whether one of them may prevail in the etiopathological onset of PARK20 and further investigation is needed in this direction.

Mitochondrial dysfunction is a common hallmark of both sporadic and genetic PD and is often associated with neuronal cell death in a number of neurodegenerative diseases (Subramaniam and Chesselet, 2013). Mitochondria are strictly connected to the ER via the mitochondrial-associated ER membranes (MAMs) through which Ca^2+^, lipids and ROS are transmitted from the ER to mitochondria (Rainbolt et al., 2014). Our findings support that the activation of PERK-CHOP pathway during chronic ER stress can even potentiate MAMs altering the mitochondrial function. Accordingly, in PARK20 cells we observed that mitochondrial alteration of MMP and morphology is dependent on PERK activation and reversed by the PERK inhibitor GSK2606414. Similar results were obtained in pink1/parkin PD models, where mitofusin contacts with damaged mitochondria sustain PERK signaling, while suppression of PERK signaling by using GSK2606414 or by genetic inhibition has a neuroprotective effect (Celardo et al., 2016), suggesting common molecular features between PARK20 and other PD types.

## Conclusion

In the present work we show that PARK20 fibroblasts display alteration of the early secretory compartments and impairment of the ER-to Golgi trafficking leading to PERK activation, OS induction and mitochondrial dysfunction. Thus, these results indicate that, beside the role of endosomal system previously shown (Fasano et al., 2018), defects of early secretory pathway could contribute to the PARK20 pathogenesis. Together with our previous findings, our data emphasize the link between membrane trafficking defects and PD. Moreover, although the correlation between mitochondrial dysfunction, OS and PERK activation in PARK20 cells needs to be further investigated, our current findings open a new lead in studying PD and phosphoinositide metabolism, providing possible novel biomarkers that can be used as diagnostic and prognostic tools for the disease.

## Data Availability

The datasets generated for this study are available on request to the corresponding author.

## Author Contributions

GA, OM, DF, and LZ performed and quantified the immunofluorescence data. MO, PDP, and RF carried out the biochemical quantitative analyses of oxidative stress. PB, MP, GDM, VB, CC, and ADR identified the patients, conducted the biopsies and provided primary cultures of fibroblasts. GA, OM, and MR analyzed UPR signaling. GA, OM, LN, and GP carried out the mitochondrial imaging analyses. EP performed electron microscopy experiments. EP and RP carried out the EM analyses. SP and PR contributed to the conception and design of the work, and wrote sections of the manuscript.

## Conflict of Interest Statement

The authors declare that the research was conducted in the absence of any commercial or financial relationships that could be construed as a potential conflict of interest.

## Supplementary Material

FIGURE S1 The exocytic trafficking is impaired in PARK20 fibroblasts. **(A)** Wild-type and PARK20 fibroblasts, grown on 60 mm dishes, were incubated in culture medium containing 0.75% serum for 7 h. Then, equal volumes of corresponding culture mediums were collected, TCA-precipitated, separated by 7.5% SDS-PAGE and revealed by western blotting with collagen IV (COL IV) antibody (right panel). 1/20 of total was analyzed by 10% SDS-PAGE and total protein secretion was revealed by Coomassie-Blue staining (left panel). **(B)** Wild-type and PARK20 fibroblasts, grown on 60 mm dishes, were biotinylated to label selectively surface proteins and cell lysates were revealed by western blotting using HRP-conjugated streptavidin.

FIGURE S2 Activation of UPR-dependent signaling pathways. **(A)** Semi-quantitative RT-PCR of Bip/Grp78 mRNA in HDF (WT) and PARK20 fibroblasts either untreated (0) or treated with 2 μg/ml Tunicamycin (TM) or 500 nM Thapsigargin (TG) for the indicated times. GAPDH mRNA was used as reference. One out of three independent experiment is shown. **(C)** Histogram shows the relative fold expression of Bip/Grp78 mRNA amplified as in **(A)** and calculated by densitometry analysis with ImageJ software. Values are expressed as mean ± SD. Controls (C) refers to untreated samples. *N* = 3. **(B)** XbpI splicing assay performed on samples treated as in **(A)**. GAPDH mRNA was used as reference. Amplicons derived from unspliced-XbpI (u) and spliced-XbpI (s) are shown. Numbers refers to the percent of spliced-XbpI to total XbpI (mean values), quantified by densitometry analysis with Image J from three independent experiments.

FIGURE S3 The figure represents the uncut or partially cut filters used to mount Figure 4A. Predicted MW of proteins are reported on the left of the panels. # Indicates filters of the same experiment stripped and re-probed with antibodies as indicated. ^∗^ Indicates the phosphorylated form of PERK.

FIGURE S4 The figure represents the uncut filter used to mount Figure 5D. Predicted MW of the HO-1 protein is reported on the left of the panel.
