# Supplementary figures and images for: PERK-Mediated Unfolded Protein Response Activation and Oxidative Stress in PARK20 Fibroblasts
Source: Front Neurosci. 2019 Jun 27;13:673. doi: 10.3389/fnins.2019.00673 (PMC6610533; doi:10.3389/fnins.2019.00673)

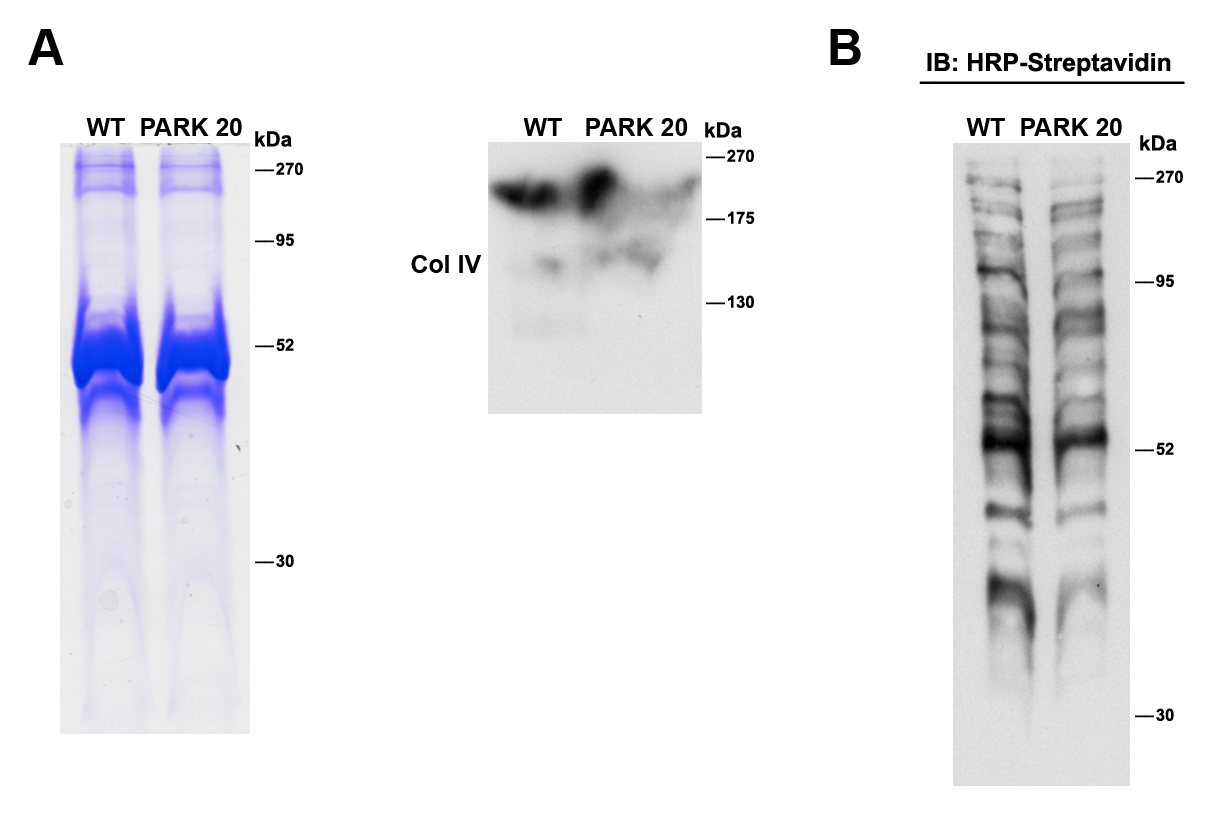

Supplement: FIGURE S1 — The exocytic trafficking is impaired in PARK20 fibroblasts. (A) Wild-type and PARK20 fibroblasts, grown on 60 mm dishes, were incubated in culture medium containing 0.75% serum for 7 h. Then, equal volumes of corresponding culture mediums were collected, TCA-precipitated, separated by 7.5% SDS-PAGE and revealed by western blotting with collagen IV (COL IV) antibody (right panel). 1/20 of total was analyzed by 10% SDS-PAGE and total protein secretion was revealed by Coomassie-Blue staining (left panel). (B) Wild-type and PARK20 fibroblasts, grown on 60 mm dishes, were biotinylated to label selectively surface proteins and cell lysates were revealed by western blotting using HRP-conjugated streptavidin. [file Image_1.TIF]

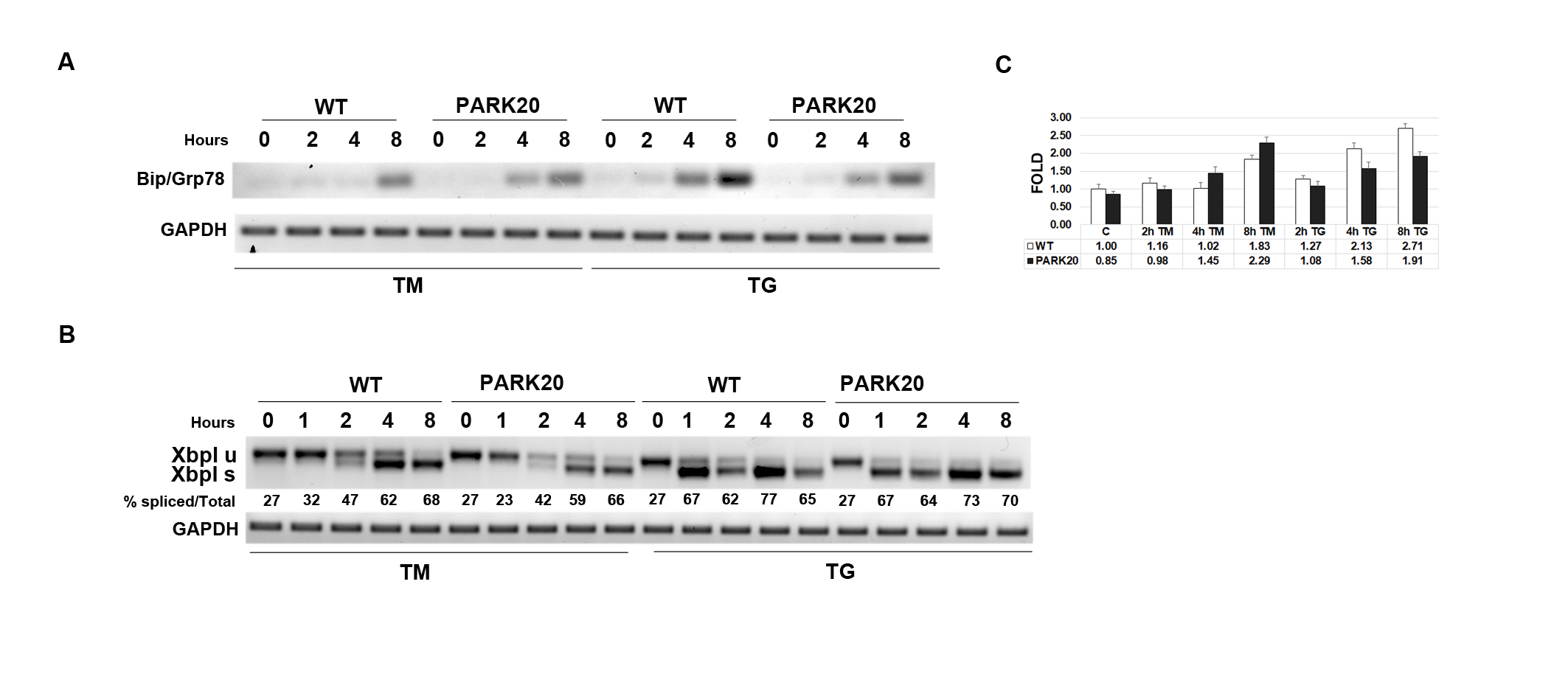

Supplement: FIGURE S2 — Activation of UPR-dependent signaling pathways. (A) Semi-quantitative RT-PCR of Bip/Grp78 mRNA in HDF (WT) and PARK20 fibroblasts either untreated (0) or treated with 2 μg/ml Tunicamycin (TM) or 500 nM Thapsigargin (TG) for the indicated times. GAPDH mRNA was used as reference. One out of three independent experiment is shown. (C) Histogram shows the relative fold expression of Bip/Grp78 mRNA amplified as in (A) and calculated by densitometry analysis with ImageJ software. Values are expressed as mean ± SD. Controls (C) refers to untreated samples. N = 3. (B) XbpI splicing assay performed on samples treated as in (A). GAPDH mRNA was used as reference. Amplicons derived from unspliced-XbpI (u) and spliced-XbpI (s) are shown. Numbers refers to the percent of spliced-XbpI to total XbpI (mean values), quantified by densitometry analysis with Image J from three independent experiments. [file Image_2.TIF]

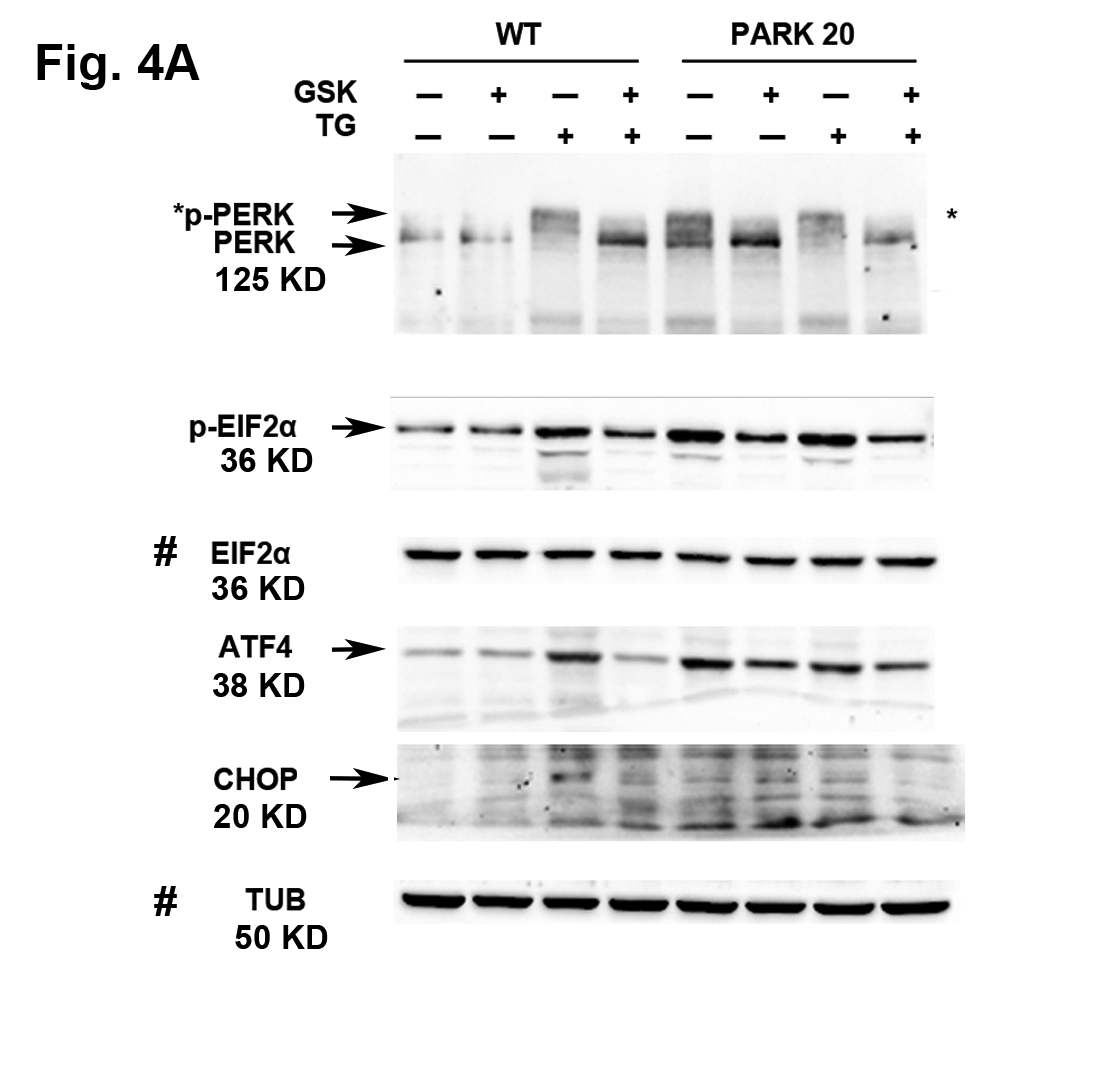

Supplement: FIGURE S3 — The figure represents the uncut or partially cut filters used to mount Figure 4A. Predicted MW of proteins are reported on the left of the panels. # Indicates filters of the same experiment stripped and re-probed with antibodies as indicated. ∗ Indicates the phosphorylated form of PERK. [file Image_3.TIF]

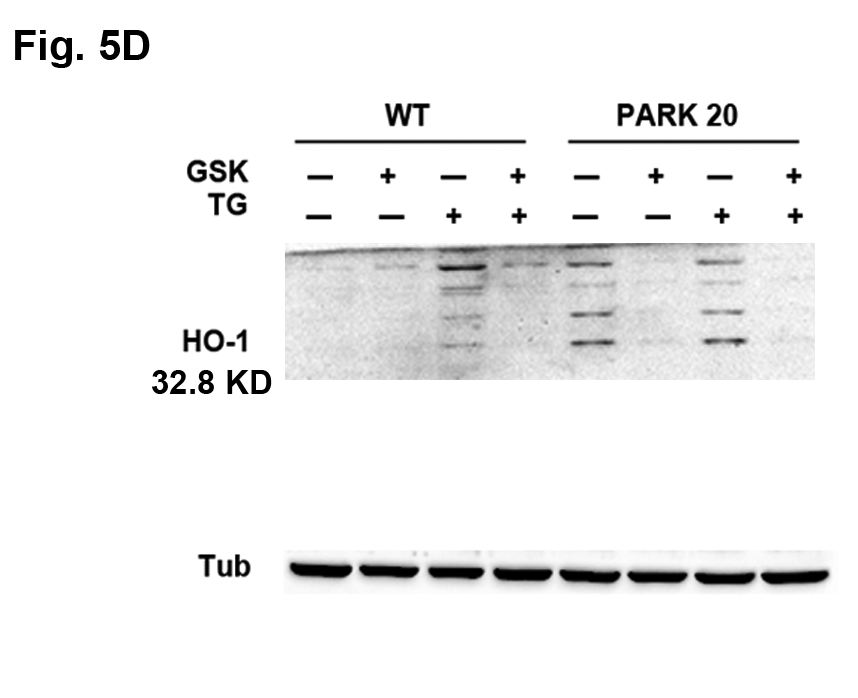

Supplement: FIGURE S4 — The figure represents the uncut filter used to mount Figure 5D. Predicted MW of the HO-1 protein is reported on the left of the panel. [file Image_4.TIF]
